# Subjective estimates of total processing time in dual-tasking: (some) good news for bad introspection

**DOI:** 10.1007/s00426-022-01762-z

**Published:** 2022-11-11

**Authors:** Daniel Bratzke, Donna Bryce

**Affiliations:** 1 Department of Psychology, University of Bremen, Bremen, Germany; 2 Department of Psychology, Eberhard Karls University of Tübingen, Tübingen, Germany

## Abstract

Previous studies have shown severe distortions of introspection about dual-task interference in the Psychological Refractory Period (PRP) paradigm. The present study investigated participants’ ability to introspect about the total trial time in this paradigm, as this temporal information may arguably be more relevant for strategic task scheduling than subjective estimates of each task within the dual task. To this end, participants provided estimates of their reaction times (IRTs) for the two subtasks in one half of the experiment, and estimates of the total trial time (ITTs) in the other half of the experiment. Although the IRT results showed the typical unawareness of the PRP effect, ITTs reflected the effects of SOA and Task 2 difficulty on objective total trial time. Additional analyses showed that IRTs were influenced by the introspective task order; that is, the ITT pattern carried over to IRTs when IRTs were assessed in the second half of the experiment. Overall, the present results show that people are able to accurately introspect about total trial time in the PRP paradigm and thus provide some good news for bad introspection in the PRP paradigm.

## Introduction

After more than a century of experimental research on human multitasking abilities, it seems fair to conclude that they are limited (for reviews see Koch et al., 2018; Pashler, 1994). One of the standard experimental paradigms that has emerged from this research tradition is the Psychological Refractory Period (PRP) paradigm (e.g., Pashler, 1994), in which participants perform two choice–reaction time tasks (Task 1 and Task 2) under different conditions of temporal overlap (i.e., different stimulus onset asynchronies, SOAs). The typical observation in this paradigm is a selective response slowing in Task 2 with increasing temporal overlap of the two tasks (i.e., the PRP effect).

Only about a decade ago, Corallo and colleagues demonstrated that introspection in the PRP paradigm is blind to the PRP effect (Corallo et al., 2008). Specifically, they observed that trialwise estimates of RT2 (introspective reaction times, IRTs) were unaffected by SOA. This basic observation has now been confirmed several times (Bratzke & Bryce, 2016; Bratzke & Janczyk, 2021; Bryce & Bratzke, 2014, 2015a, 2017; Marti et al., 2010). This apparent unawareness of multitasking costs has been regarded as evidence for a central processing bottleneck that encompasses response selection and conscious perception (Corallo et al., 2008; Marti et al., 2010). However, results from a recent introspective PRP study, which used a timeline method to assess the full subjective time course of a PRP trial, raise doubts about this interpretation (Bryce & Bratzke, 2022). In this study, unawareness of the PRP effect seemed to depend on the stimulus modality order of the two tasks, with an unawareness in auditory-visual and an awareness in visual–auditory dual tasks. This observation is clearly inconsistent with the idea of an amodal conscious perception bottleneck and hints at a memory-related source of the introspective blind spot.

To our knowledge, all previous studies on introspection about dual-task performance using the “standard” visual analog scale (VAS) method assessed introspective RTs either for both tasks separately or only for Task 2. None of these studies, however, asked participants to estimate the total processing time of a PRP trial.^1^ Previous models of task organization in the multitasking context have considered the objective time demands of to-be-scheduled tasks as an important factor in task organization (e.g., Salvucci, 2005). If a dual task needs to be scheduled within a larger overarching multitasking context, the total time demands to complete both tasks may be just as important as, if not more important than, the time demands of each separate task. Furthermore, when it comes to task scheduling, our subjective representation of the time taken may be more influential than the objective time required and prior research has demonstrated that these can differ considerably from each other.

One could argue that a subjective estimate of total processing time can be reconstructed from the IRTs and the objective or estimated SOA and indeed Marti et al. (2010) did take such an approach. The results of Marti et al.’s (2010) reconstruction of the subjective phenomenology of PRP trials suggest that participants can track the effect of SOA on total trial duration; that is, that total trial duration increased with increasing SOA. However, it is not clear whether such a reconstructed estimate equals the actual subjective experience of total trial duration. In a previous study, we observed that participants were much better at reconstructing the temporal course of a PRP trial when they indicated the trial events (i.e., stimulus and response onsets in the two tasks) on a common timeline instead of providing separate RT estimates (Bryce & Bratzke, 2017). This suggests that it may be especially difficult to extract the time intervals of interest (i.e., RT1, RT2, SOA) from the subjective time course of a PRP trial. In the present study, we therefore examined whether participants can accurately introspect about the total time demands of a dual task when they are asked to provide a direct and explicit estimate of total trial time in the PRP paradigm with the standard visual–auditory modality order. As in previous studies, we also manipulated the perceptual difficulty of Task 2 to test whether participants are sensitive to the rather small effects of Task 2 difficulty on total trial time as well as on RT2.

## Methods

### Participants

Sixteen volunteers participated for monetary compensation or course credit (one participant was excluded and replaced by another participant because of not adhering to the instruction, i.e., not moving a marker on 78% of estimates). This sample size was chosen to achieve a statistical power that at least equaled those of previous introspective RT studies (*N* = 13 in Corallo et al., 2008; *N* = 10 in Marti et al., 2010; *N* = 16 in Bryce & Bratzke, 2014, 2017, 2022). The final sample had a mean age of 22.4 years (11 female). All participants reported normal or corrected-to-normal vision, were naïve regarding the underlying hypotheses, and provided written informed consent prior to data collection.

### Apparatus and stimuli

The experiment was programmed in Matlab using the Psychophysics Toolbox (Version 3.0.8; Brainard, 1997; Kleiner et al., 2007) and controlled by an Apple Mac Pro Computer connected to a 17-inch CRT monitor. The responses in Task 1 (R1) and Task 2 (R2) were collected with two custom-built response boxes placed to the left and right of the participant on the table that were operated with the left and right index and middle fingers. Task 1 stimuli (S1) were a 440 Hz (low-pitched) or a 880 Hz (high-pitched) tone presented through headphones at an intensity of 60 dB(A). Task 2 stimuli (S2) were a plus (+) and a minus (−) symbol, which were degraded by a square area of a random dot pattern. There were 6 levels of degradation for each symbol (25–275 dots, in steps of 50; see Bryce & Bratzke, 2014, 2017). Visual stimuli were presented in white against a black background. The visual analog scales to assess RT and total trial time estimates consisted of a horizontal line with seven equally spaced vertical ticks (major ticks at both ends and at the center and minor ticks in between). For RT estimates, the major ticks of the VAS were labeled “0 ms”, “750 ms” and “1500 ms”; for total trial estimates the two ends were labeled “0 ms” and “4000 ms”. To indicate their estimates, participants moved a small rectangular marker (initially presented at the center position) with the “1” key (marked with a left-pointing arrow) and the “3” key (marked with a right-pointing arrow) of the numeric pad of the computer keyboard. The final position was confirmed with the space key.

### Tasks and procedure

Participants started a trial with a keypress of one of the four response keys situated on the custom-built response boxes. First, a central fixation point appeared for 250 ms. Then, the tone (S1) was presented for 150 ms. Next, the symbol (S2) appeared in the center of the screen according to a variable SOA (50, 250, 1250 ms). Participants were instructed to provide a response to the tone with their left hand (low-pitched tone—index finger, high-pitched tone—middle finger) and to the symbol with their right hand (minus symbol—index finger, plus symbol—middle finger). Written instructions emphasized both speed and accuracy. In case of an error in at least one of the two tasks, participants received an error message for 500 ms. They received an additional feedback message (500 ms), if the inter-response interval was shorter than 100 ms, displaying a reminder that both responses should be provided as fast and as accurately as possible. Participants were then presented with the visual analog scale and asked to estimate their RTs (from stimulus onset until the corresponding response for each task) or the total trial time (from S1 onset until the last response). In trials with RT estimation, separate visual analog scales appeared for IRT1 and for IRT2 with a 500 ms blank interval between them.

Participants estimated their RTs and the total trial time in different halves of the experiment, with the order counterbalanced across participants. The experiment started with a short familiarization block of 9 trials, in which participants performed only the PRP task without any time estimation. There was another short practice block of 9 trials at the beginning of each half of the experiment, in which participants performed the PRP and the respective time estimation task. In each half of the experiment, these practice trials were followed by 144 experimental trials divided into 4 blocks of 36 trials each. The 144 trials resulted as the combination of 2 S1 (440 vs. 880 Hz) × 12 S2 (minus vs. plus sign, each with 6 levels of degradation) × 3 SOAs (50, 250, and 1250 ms), repeated twice. Accordingly, there were 288 experimental trials in total.

### Design and analyses

As in previous studies (Bryce & Bratzke, 2014, 2017), the 6 levels of S2 degradation were classified into 2 levels of Task 2 difficulty (easy vs. hard). For analyses of objective and introspective RTs and total trial times, only trials in which both responses were correct were included, and trials were excluded as outliers if either RT1 or RT2 deviated more than 3.0 *SD*s from the respective cell mean (calculated separately for each participant, SOA and Task 2 difficulty level). Furthermore, only trials with an inter-response interval larger than 100 ms were included in these analyses to exclude an influence of response grouping on the result pattern (see Ulrich & Miller, 2008). Separate ANOVAs with the within-subjects factors SOA, Task 2 difficulty, and introspective task (IRT vs. ITT) were conducted for reaction times (RT1 and RT2), error rates (ER1 and ER2) and objective total trial time (TT). Another set of ANOVAs with the within-subjects factors SOA and Task 2 difficulty was conducted for introspective reaction times (IRT1 and IRT2), and introspective total trial time (ITT). To directly compare introspection between RT2 and TT, a combined ANOVA was conducted with the within-subject factors SOA, time interval (RT2 vs. TT), and measure (subjective vs. objective).^2^ Additionally, we calculated Pearson correlations and linear regressions between objective and introspective measures (RT2/IRT2 and TT/ITT) per participant (aggregated across SOA and Task 2 difficulty levels; see also Bryce & Bratzke, 2015a; Corallo et al., 2008).

Our hypotheses were as follows. We expected to observe the typical objective RT pattern in the PRP paradigm as predicted by the central bottleneck model (e.g., Pashler, 1994), that is, increasing RT2 with decreasing SOA (i.e., the PRP effect), and no SOA effect on RT1. Based on a wealth of introspective PRP studies using an auditory–visual task order, we expected introspective RTs to show the typical unawareness of the PRP effect, that is, IRT2 as well as IRT1 should be unaffected by SOA. Regarding total trial time, objective total trial time should increase with increasing SOA and we expected this effect to be reflected in ITT. The factor of Task 2 difficulty (i.e., perceptual difficulty) should selectively affect Task 2 performance, and the effect should be absorbed at short SOAs since it asserts its effect in the perceptual processing stage (reflected in an under-additive interaction between Task 2 difficulty and SOA on RT2; e.g., Bryce & Bratzke, 2014; Pashler, 1994). The same interaction pattern would be expected to show up in objective total trial time. Based on previous results (Bryce & Bratzke, 2014; Corallo et al., 2008), we hypothesized that this interaction pattern would be reflected in IRT2 and also in ITT.

## Results

For all analyses except analysis of error performance, 15.2% error trials (4.6% with errors only in Task 1, 9.5% with errors only in Task 2, and 1.0% with errors in both tasks) were excluded, and 3.2% of correct trials were excluded as RT outliers. A further 0.9% of the remaining trials were excluded as grouped trials.

### Task 1

**Fig. 1:**
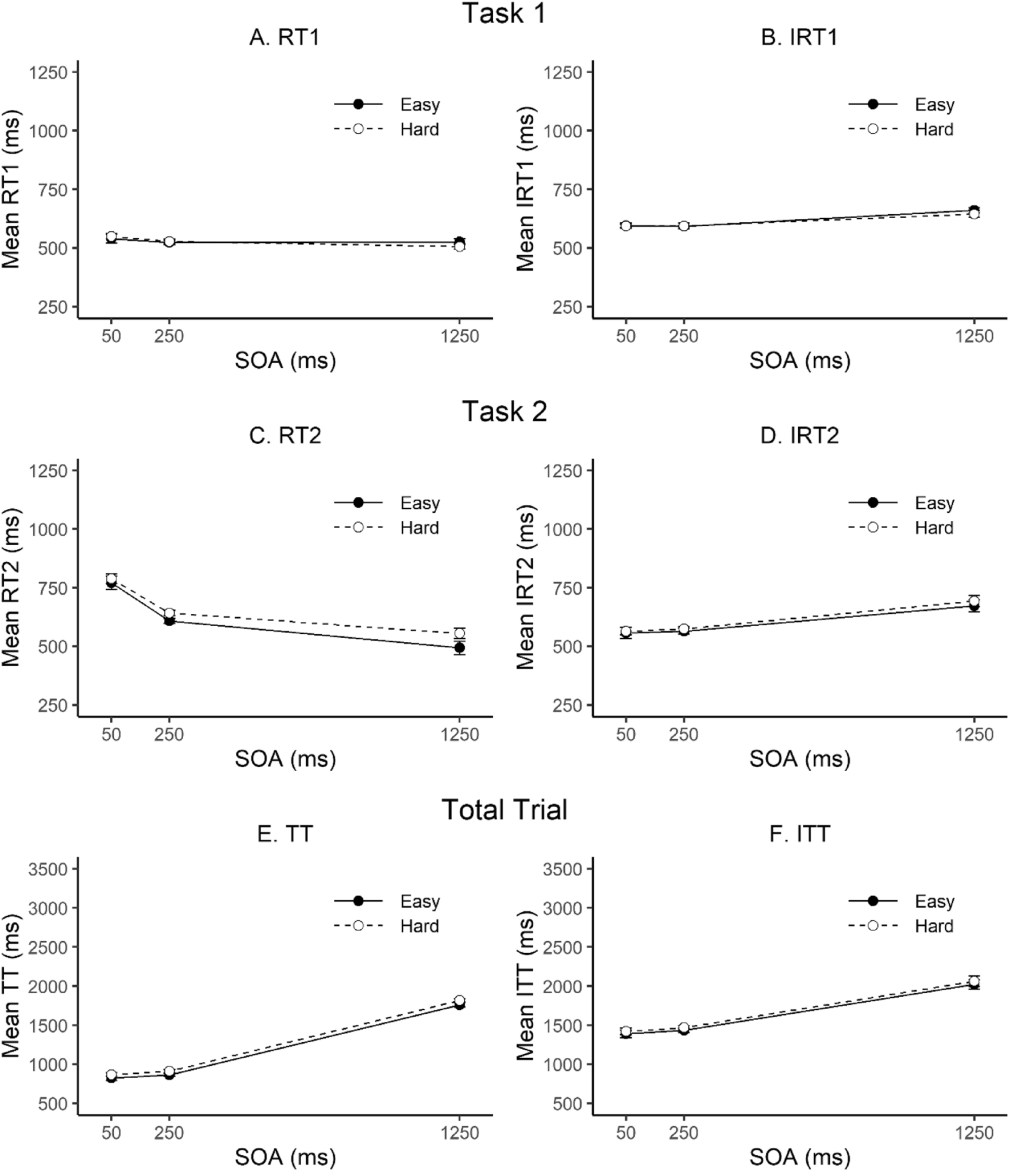
Mean reaction and total trial times (left column; RT/TT), and introspective reaction and total trial times (right column; IRT/ITT) as a function of SOA and Task 2 difficulty. Note that the scaling of the y-axis is compressed by the factor 3 for total trial measures. Error bars represent ± 1 within-subjects *SE* according to Morey (2008)

As can be seen in panel A of Fig. 1, RT1 was rather constant across SOAs and there was only very little variation of RT1 depending on Task 2 difficulty. Accordingly, the ANOVA revealed no significant effects on RT1, all *p*s ≥ 0.159. In contrast, IRT1 was significantly affected by SOA, *F*(2, 30) = 11.82, *p* = 0.001, *η*_p_^2^ = 0.44, with an increase of IRT1 at the longest SOA (653 ms vs. 594 ms at the 50 ms SOA and 593 ms at the 250 ms SOA; see panel B of Fig. 1). The main effect of Task 2 difficulty and the two-way interaction were not significant, both *F*s < 1. There was also a significant SOA effect on Task 1 error rates, *F*(2, 30) = 6.75, *p* = 0.009, *η*_p_^2^ = 0.31. Participants made slightly more errors at the short SOA (7.8%) than at the middle (4.6%) and the long SOA (4.4%). All other main and interaction effects on Task 1 error rate were not significant, all *p*s ≥ 0.109.

### Task 2

RT2 performance (panel C of Fig. 1) showed a PRP effect of 255 ms, *F*(2, 30) = 36.18, *p* < 0.001, *η*_p_^2^ = 0.71. There was also a significant Task 2 difficulty effect on RT2 (38 ms), *F*(1, 15) = 13.82, *p* = 0.002, *η*_p_^2^ = 0.48. In contrast to the prediction of the central bottleneck model (i.e., an under-additive interaction between SOA and perceptual task difficulty; e.g., Pashler, 1994), there was no significant interaction between SOA and Task 2 difficulty, *F*(2, 30) = 1.53, *p* = 0.234, *η*_p_^2^ = 0.09, even though the RT2 pattern is numerically consistent with the predicted under-additivity (the Task 2 difficulty effect is 62 ms at the long SOA vs. 22 ms at the short SOA). All other main and interaction effects on RT2 were also not significant, *p*s ≥ 0.343. Unexpectedly, IRT2 was also significantly affected by SOA, *F*(2, 30) = 10.92, *p* = 0.002, *η*_p_^2^ = 0.42. However, as can be seen in panel D of Fig. 1, this main effect of SOA reflects an increase of IRT2 at the long SOA (683 ms vs. 570 ms at the middle and 561 ms at the short SOA) rather than the objective PRP effect. There was neither a main effect of Task 2 difficulty on IRT2, *F*(1, 15) = 2.29, *p* = 0.151, *η*_p_^2^ = 0.13, nor an interaction of SOA and Task 2 difficulty, *F*(2, 30) = 0.28, *p* = 0.758, *η*_p_^2^ = 0.02. Task 2 error rates were only affected by Task 2 difficulty, *F*(1, 15) = 54.51, *p* < 0.001, *η*_p_^2^ = 0.78, all other *p*s ≥ 0.136. As one would expect, participants made more errors when Task 2 was difficult than easy (15.1% vs. 6.1%).

### Total trial

Objective and introspective total trial times are depicted in the lower part of Fig. 1 (panels E and F). Objective trial time (TT) was affected by SOA, *F*(2, 30) = 622.61, *p* < 0.001, *η*_p_^2^ = 0.98, and by Task 2 difficulty, *F*(1, 15) = 13.85,* p* = 0.002, *η*_p_^2^ = 0.48. All other main and interaction effects were not significant, *p*s ≥ 0.235. Even though the effects on introspective total trial time (ITT) were smaller in absolute terms, they largely mirrored the objective result pattern. Accordingly, ITT was significantly affected by SOA, *F*(2, 30) = 47.24, *p* < 0.001, *η*_p_^2^ = 0.76, and by Task 2 difficulty, *F*(1, 15) = 4.96, *p* = 0.042, *η*_p_^2^ = 0.25, and the two-way interaction was not significant, *F* < 1.

### Task 2 vs total trial

The combined ANOVA with the factors time interval (RT2 vs. TT), SOA, and measure (objective vs. introspective) revealed a significant three-way interaction, *F*(1, 15) = 38.1, *p* < 0.001, *η*_p_^2^ = 0.72, confirming the difference in the result pattern between RT2 and TT. In a next step, we conducted separate ANOVAs for the two intervals, that is, RT2 and TT. In both ANOVAs, the two-way interaction between SOA and measure was significant (RT2: *F*(1, 15) = 42.98, *p* < 0.001, *η*_p_^2^ = 0.74; TT: *F*(1, 15) = 18.76, *p* < 0.001, *η*_p_^2^ = 0.56). The significant interaction for RT2 confirmed that while objective RT2 decreased with increasing SOA, IRT2 increased with increasing SOA. The significant interaction for TT reflected that participants underestimated the objective SOA effect on total trial time (see panels E and F of Fig. 1).

The mean correlation between subjective and objective measures was higher for total trial time (*M* = 0.70, *SD* = 0.14) than for RT2 (*M* = 0.24, *SD* = 0.21), *t*(15) = 9.44, *p* < 0.001. Similarly, the mean slope of the individual regressions was steeper and closer to 1 for total trial time (*M* = 0.63, *SD* = 0.36) than for RT2 (*M* = 0.19, *SD* = 0.23), *t*(15) = 6.23, *p* < 0.001.

### Introspective task order

**Fig. 2:**
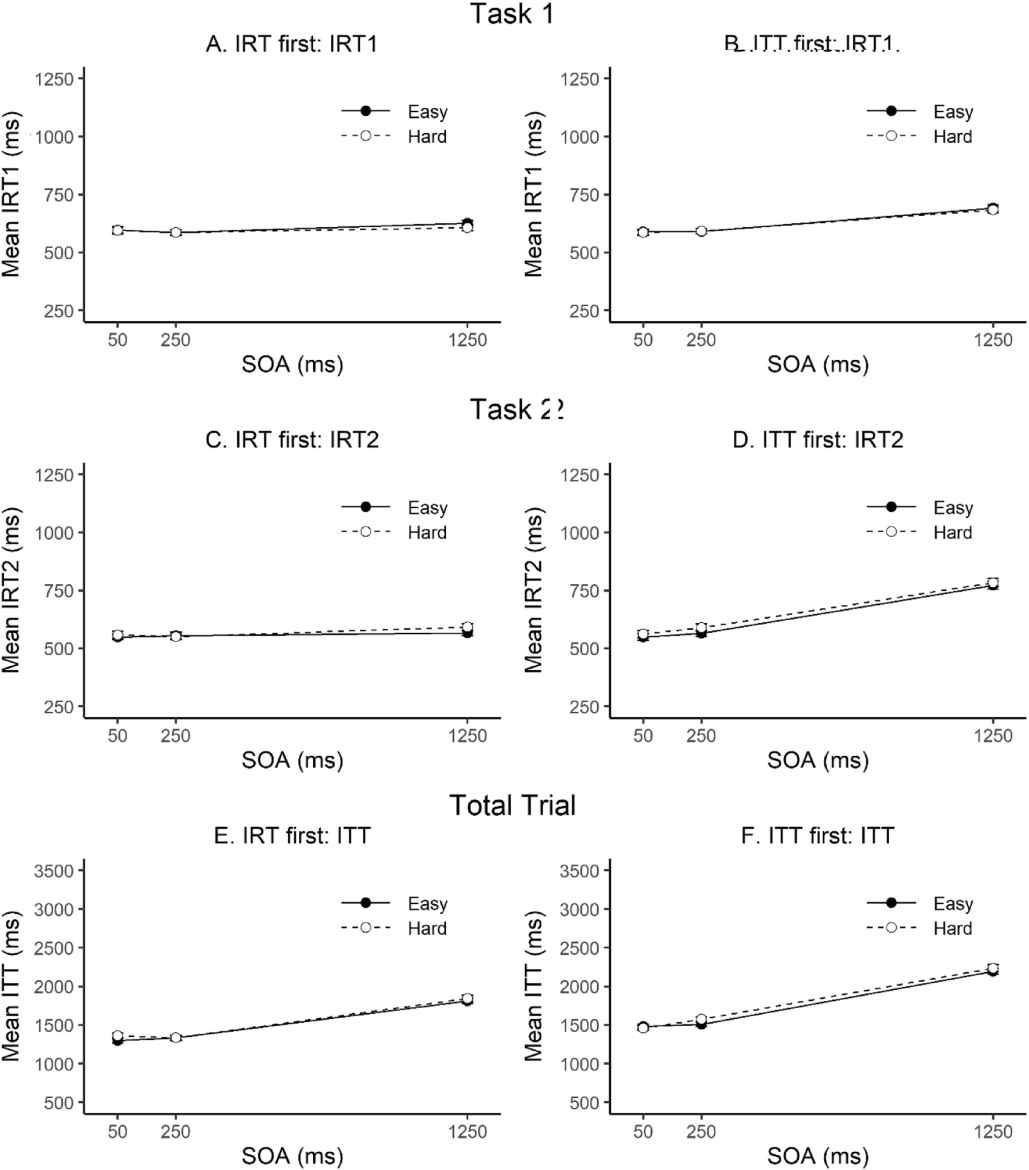
Mean introspective reaction times (IRT) in Task 1 and Task 2, and mean introspective total trial times (ITT) as a function of SOA, Task 2 difficulty and introspective task order (ITT first vs. IRT first). Note that the scaling of the y-axis is compressed by the factor 3 for total trial measures. Error bars represent ± 1 within-subjects *SE* according to Morey (2008)

Since an increase in IRT with increasing SOA was not hypothesized nor reflected in objective RTs, and total trial times did show such an effect, we conducted a follow-up analysis to explore whether introspective task order might have affected objective and/or introspective performance. We repeated all ANOVAs for Task 1, Task 2 and TT with the additional between-subjects factor introspective task order (IRT first vs. ITT first). Only main effects and interactions including introspective task order are reported in the following. There was no significant main effect of task order nor any interactions including task order on objective task performance (RT1, RT2 and TT), all *p*s ≥ 0.074. However, introspective reports were significantly affected by order (see right panels of Fig. 2). Introspective task order modulated the effect of SOA on IRT1, *F*(2, 28) = 6.34, *p* = 0.005, *η*_p_^2^ = 0.31. Separate ANOVAs for the different orders revealed that the effect of SOA on IRT1 was only significant in the ITT first order. The SOA effects (long–short SOA) on IRT1 were 99 ms in the ITT first order (*p* < 0.001, *η*_p_^2^ = 0.75) and 19 ms in the IRT first order (*p* = 0.308, *η*_p_^2^ = 0.15). A similar modulation of the SOA effect by order was observed for IRT2, *F*(2, 28) = 11.61, *p* = 0.002, *η*_p_^2^ = 0.45. Again, the SOA effect was only significant in the ITT first order. The SOA effects on IRT2 were 222 ms in the ITT first order (*p* < 0.001, *η*_p_^2^ = 0.75) and 21 ms in the IRT first order (*p* = 0.448, *η*_p_^2^ = 0.09). Regarding ITT, there was only a significant three-way interaction of task order, SOA, and Task 2 difficulty (see panels E and F of Fig. 2), *F*(2, 28) = 3.45, *p* = 0.046, *η*_p_^2^ = 0.20, all other *p*s ≥ 0.187.

## Discussion

In the present study, we aimed to uncover whether people can introspect about their total processing time in a standard dual-task situation, namely the Psychological Refractory Period paradigm. The data show that participants were able to report the effects of SOA and Task 2 difficulty on total trial time despite being unaware of the PRP effect when RTs are estimated for each task separately. These results confirm in a more direct way the implication of previous results by Marti et al. (2010) that the subjective phenomenology of dual-task trials tracks the effects of SOA on total trial time.

The present results again replicated the typical introspective blind spot of the PRP effect that is observed when participants process an auditory Task 1 and visual Task 2 and then provide their IRTs via a VAS (for a recent report of notably spared introspection in the visual–auditory task order using a timeline method, see Bryce & Bratzke, 2022). Nevertheless, the present IRT results also showed some deviations from the typical pattern; that is, both IRTs increased with increasing SOA. Importantly, none of the objective RTs showed a corresponding result pattern. Since both the objective and introspective total trial times did show such an effect, we conducted a follow-up analysis including introspective task order. This analysis revealed that SOA effects on each IRT were modulated by task order, again without corresponding effects on objective RTs. This suggests that experience with the estimation of total trial time affected the IRTs participants provided. Participants who performed the estimation of total trial time in the first half of the experiment showed a tendency to report a similar SOA pattern also in their IRTs during the second half of the experiment. Since ITTs and IRTs were never assessed within the same trial (and also not within the same half of the experiment), this carry-over effect must reflect a long-term effect rather than a short-lived response bias (e.g., a within-trial anchoring effect). We therefore interpret it as an example of cue utilization in introspection about multitasking performance. According to this view, participants use various cues to infer their RTs, for example, the feeling of difficulty or other temporal intervals present in the trial (see Bratzke & Bryce, 2016, 2019; Bratzke et al., 2014; Bryce & Bratzke, 2014). Importantly, some of these cues may be invalid. In the present experiment, we posit that participants wrongly inferred longer RTs in long than short SOA trials based on the insight they previously gained when estimating total trial times (i.e., longer total trial times in long than short SOA trials). While the results of the order analysis need to be interpreted with caution due to the halved sample size in the order analysis, this finding constitutes further evidence that IRTs collected in this manner are unstable and particularly vulnerable to bias.

In contrast, the total trial time estimates appear more accurate. Importantly, this was confirmed when the two time intervals (RT and TT) and measures (subjective and objective) were analyzed in a combined ANOVA. But how accurate is introspection about total trial time? First, while the predicted under-additive interaction between SOA and Task 2 difficulty on RT2 as well as on ITT was not significant, estimates of total trial time showed the same qualitative result pattern as objective total trial times. That is, they both showed an increase with increasing SOA, a Task 2 difficulty effect and no two-way interaction. Second, correlational and regression analyses showed that the relationship between subjective and objective measures was much stronger for total trial time than for RT2. While one could argue that participants overestimated total trial time at short SOA (see lower part of Fig. 1), we caution against interpretations based on absolute ITT values, since these are greatly determined by the rather arbitrary labels of the VASs. In sum, it seems fair to us to conclude that estimates of total trial time rather accurately (even though not perfectly) tracked the effects of SOA and Task 2 difficulty on objective total trial time.

A potential limitation of the experimental design deserves some consideration, namely the fact that in ITT blocks participants provided only one estimate whereas in IRT blocks they provided two estimates. One could question whether the reduced cognitive load in the ITT blocks is responsible for the superior introspective accuracy observed. There is indeed evidence from time perception literature that timing of multiple intervals can affect timing precision and accuracy (Brown & West, 1990; Bryce & Bratzke, 2015b, 2016; van Rijn & Taatgen, 2008). Previous introspective PRP studies, however, have demonstrated that dual timing is not the cause of the introspective blind spot in the PRP paradigm. For example, in Bryce and Bratzke (2014), the same null effect was observed although IRT2 and IRT1 were assessed in separate halves of the experiment. In a study using the method of constant stimuli, in which participants only compared RT2 to presented intervals, the same unawareness of the PRP effect was observed (Bratzke & Bryce, 2016); in Experiment 1B of Bryce and Bratzke (2022) participants only reported the events related to Task 2 on the timeline and also did not report a PRP effect. Furthermore, the fact that selectively the PRP effect is not reflected in IRT2, while other effects of Task 2 manipulations are usually reflected, further argues against the dual timing explanation.

Given the present dissociation in the accuracy of different introspective estimates in the multitasking context, it seems pertinent to ask whether introspections about the time demands of task processing could actually impact on people’s behavior in terms of strategic task scheduling. A related theoretical framework is the optimization account by Miller and colleagues, which assumes that both parallel and serial processing are possible in dual-task processing, and that the processing mode is chosen to optimize the total sum of RTs (Miller et al., 2009). With respect to broader multitasking contexts, computational cognitive architectures like ACT-R (e.g., Anderson et al., 2004) and EPIC (e.g., Meyer & Kieras, 1999) have been used to model task organization. As in the optimization account, in these modeling frameworks the time demands of to-be-scheduled tasks play an important role in task organization (e.g., Salvucci, 2005). However, usually the objective rather than the subjective time demands are considered in these models. We believe that it is important to consider also the subjective time demands of a task, as they can considerably differ from the objective ones. More specifically, according to the present results, subjective task demands of the total trial time may be a reliable source of information, whereas subjective time demands of each separate sub-task may not.

There is a rich literature on the relationship between metacognitive monitoring and cognitive control, suggesting an important functional role of metacognitive experience in behavioral control in higher-level cognitive tasks like studying for an exam or reasoning (e.g., Metcalfe, 2009, Thompson et al., 2011). To our knowledge, however, only very few studies have investigated how subjective experience, which might differ from objective performance as in the PRP paradigm, impacts on behavioral adjustments at the micro-level of standard experimental multitasking paradigms. A related study by Desender and colleagues (2014) using a masked priming paradigm provided evidence that subjective rather than objective conflict (i.e., incongruence between prime and target) triggered conflict adaptation in subsequent trials (but see Abrahamse & Braem, 2015, for criticism of Desender et al.’s interpretation and Foerster et al., 2017, for a replication failure). Such sequential modulations of task performance are not unique to single-task performance and have also been observed in dual tasks (Janczyk, 2016; Olszanowski et al., 2015; Strobach et al., 2021). The investigation of sequential modulations in dual-task paradigms may thus provide a starting point for further research regarding the role of introspection in behavioral adaptations in this context (see also Bratzke & Janczyk, 2021).

Another related paradigm at the other pole of the multitasking continuum (i.e., switching between tasks without temporal overlap) is the voluntary task-switching paradigm, in which participants can freely choose which of two tasks to perform. Usually a strong tendency to repeat a task instead of switch between tasks is observed (e.g., Arrington & Logan, 2005). Two previous observations suggest that introspection might play an important role in this repetition bias. Namely, people seem to be aware of their switch costs (Bratzke & Bryce, 2019﻿, 2022), and they tend to switch to another task when the SOA between the new and the old task corresponds to their switch costs (e.g., Mittelstädt et al., 2018). These observations clearly show that task-switching behavior depends on switch costs; however, it is difficult to distinguish between the role of introspection and objective time demands in voluntary task-switching as introspective and objective RTs are highly correlated in task-switching (see Bratzke & Bryce, 2019).

In the present study, introspection about total trial time biased introspection of RT2, but only if attention was drawn toward this time interval by instruction (i.e., asking participants for estimates of total trial time). Thus, introspection about total trial time is probably not what participants do spontaneously in this context, at least when they are asked to introspect about RT1 and/or RT2 at the same time. This raises the questions of what kind of information or processes participants actually introspect about when engaging dual-task processing, and how overarching performance goals (e.g., optimization of task-scheduling) would affect their introspective ‘focus’. Furthermore, the subjective time demands of task processing are certainly only one aspect of introspective and metacognitive experiences during such a task. Which of these experiences arise under which conditions and how they are functional for cognitive and behavioral control remain important questions for future research.

In conclusion, the present study investigated participants’ ability to introspect about the total time taken to process two tasks in the PRP paradigm. The results showed that estimates of total trial time rather accurately tracked the effects of SOA and Task 2 difficulty on objective total trial time, although estimates of each RT reflected unawareness of the PRP effect. The present results thus provide some good news for bad introspection in dual-tasking. Furthermore, the present carry-over effects from estimation of total trial times to RT estimation provide further evidence that introspective RTs are oftentimes biased by (or even based on) potentially invalid cues.

## Data Availability

The raw data are available at https://osf.io/9qmn8/?view_only=55ad07f723d94d248000c594b7087d6c.

## Footnotes

1 Bryce and Bratzke (2017, 2022) collected estimates of total trial duration in introspective PRP studies using a timeline method (i.e., after participants had recreated the time course of a PRP trial on a timeline). These estimates, however, were not analyzed because participants reported very little confidence in these ratings, as the information appeared to them as being already very much degraded after recreating the PRP trial.

2 We thank Jérôme Sackur for suggesting this analysis.
